# Dietary Diversity and Meal Frequency Practices among Infant and Young Children Aged 6–23 Months in Ethiopia: A Secondary Analysis of Ethiopian Demographic and Health Survey 2011

**DOI:** 10.1155/2013/782931

**Published:** 2013-11-24

**Authors:** Melkam Aemro, Molla Mesele, Zelalem Birhanu, Azeb Atenafu

**Affiliations:** ^1^DKT Ethiopia, Addis Ababa, Ethiopia; ^2^Department of Human Nutrition, Institute of Public Health, University of Gondar, P.O. Box 196, Gondar, Ethiopia; ^3^Department of Reproductive Health, Institute of Public Health, University of Gondar, P.O. Box 196, Gondar, Ethiopia

## Abstract

*Background*. Appropriate complementary feeding practice is essential for growth and development of children. This study aimed to assess dietary diversity and meal frequency practice of infants and young children in Ethiopia. *Methods*. Data collected in the Ethiopian Demographic and Health Survey (EDHS) from December 2010 to June 2011 were used for this study. Data collected were extracted, arranged, recoded, and analyzed by using SPSS version 17. A total of 2836 children aged 6–23 months were used for final analysis. Both bivariate and multivariate analysis were done to identify predictors of feeding practices. *Result*. Children with adequate dietary diversity score and meal frequency were 10.8% and 44.7%, respectively. Children born from the richest households showed better dietary diversity score (OR = 0.256). Number of children whose age less than five years was important predictor of dietary diversity (OR = 0.690). Mothers who had exposure to media were more likely to give adequate meal frequency to their children (OR = 0.707). *Conclusion*. Dietary diversity and meal frequency practices were inadequate in Ethiopia. Wealth quintile, exposure to media, and number of children were affecting feeding practices. Improving economic status, a habit of eating together, and exposure to media are important to improve infant feeding practices in Ethiopia.

## 1. Background

Insufficient quantities and inadequate quality of complementary foods, poor child feeding practices, and high rates of infections have a detrimental effect on health and growth in children less than 2 years of age. Even with optimum breastfeeding, children will become stunted if they do not receive sufficient dietary diversity and meal frequency after 6 months of age [1, 2].

Any damage caused by nutritional deficiencies during the first two years of life could lead to impaired cognitive development, compromised educational achievement, and low economic productivity [3–5]. Infant malnutrition results in growth retardation and smaller adult stature and also is correlated with inadequate immune response and increased risk of childhood mortality [6].

An estimated 6% of under-five deaths can be prevented by ensuring optimal complementary feeding among which dietary diversity and meal frequency are the most important ones, significantly contributing to the realization of Millennium Development Goal 4 [7].

Measures of infant and child nutritional status suggest that rates of malnutrition increase markedly between 4 and 12 months of age, around the time that infants begin to receive complementary foods in addition to breast milk [8–10]. According to the Ethiopian Demographic and Health Survey 2000, 43% of infants aged 6–9 months receive solid food in addition to breast milk the rest 57% who are not given complementary food are more prone to stunting and wasting [11].

In Ethiopia, only 50% of infants aged 6–8 months receive complementary foods while continuing to be breastfed [12]. These data, however, do not reflect the quality of the complementary foods received. Meeting minimum standards of dietary quality is a challenge in many developing country settings including Ethiopia, especially in areas where household food security is poor, and it has often not been given enough emphasis. Children may not be fed frequently enough during the day, or the quality of the food may be inadequate [13, 14].

## 2. Methods

Data is collected on a total number of 2836 infant and young children aged between 6–23 months from the selected households by Ethiopian Demographic and Health Survey 2011. All the samples are used for the proposed study. Data was extracted from the Ethiopian Demographic and Health Service (DHS) 2011 by downloading from Measure DHS website in SPSS format after obtaining permission from ORC Macro. Further data cleaning was done by the investigators on a total of 2836 infant and young children included in our study. Information on a wide range of potential independent variables (socio-demographic, mothers, household, child, and health care characteristics) is extracted accordingly.

The 2011 EDHS samples were selected using a stratified, two stage cluster design, and enumeration areas were the sampling units for the first stage. Data were collected from households which comprised secondary sampling unit. The data are rearranged and recoded by the investigators using SPSS version 17. To avoid problems that could arise from the nonproportional distribution of the clusters by region and to make the regional distribution nationally representative, all the data in the descriptive statistics part were weighted and descriptive statistics and cross tabs were analyzed. Tables and graphs are used for data presentation. Binary logistic regression is employed to explore association between dependent variable and a wide range of independent variables. Variables with *P* value ≤0.2 entered multivariate logistic regression which controls the undesirable effects of confounding variables. Variables with *P* value of less than 0.05 were considered as significant predictors. The result was presented with odds ratio (OR) and 95% confidence interval (CI).

### 2.1. Operational Definitions

*(1) Minimum Dietary Diversity.* It is proportion of children with 6–23 months of age who received foods from four or more food groups of the seven food groups. 

*(2) Minimum Meal Frequency.* Proportion of breastfed and nonbreastfed children aged 6–23 months who received solid, semisolid, or soft foods (but also including milk feeds for non-breastfed children) with the minimum number of 2 and 3 times for those breastfed infants and children aged 6–23 months and 4 times for nonbreastfed infants and children aged 6–23 months, respectively. 

*(3) Adequate Dietary Diversity.* Infant and children with 6–23 months of age got MDD. 

*(4) Adequate Meal Frequency.* Infant and children with 6–23 months of age got MMF. 

(5) *Satisfactory Exposure to Media*. Women aged 15–49 years at least once a week read a newspaper or magazine or listen to radio, or watche television. 

Ethical clearance was first obtained from the Institutional Ethical Review Board of Institute of Public Health, College of Medicine and Health Sciences, University of Gondar. The analyses of this paper are confined to secondary data, approval to analyze the secondary data is requested from the CSA or ORC Macro (Demographic and Health Survey) and we have been authorized to download data from the Demographic and Health Surveys (DHS) online archive.

## 3. Result

### 3.1. Sociodemographic Characteristics

A total of 2836 children were included in the analysis among which 1059 (37.4%) were in the age group 6–11 months, 943 (33.2%) in 12–17, months and the rest 834 (29.4) found in 18–23 months. The mean age of children was 1.16 (±0.43 years). The study revealed that majority of the mother (67.5%) had no education.

Two thirds (66.8%) of the mothers were not working at the time of data collection. Of the total households, 24.1% were found in the poorest wealth quintile. The present study also showed that only 22.5% had satisfactory exposure to media and most of the participants (87%) were from rural areas (Table 1).

### 3.2. Dietary Diversity and Meal Frequency Practice

The proportion of children with adequate dietary diversity in study was 10.8%. Nearly half of children (44.7%) practiced insufficient meal frequency for complementary foods.

### 3.3. Factors Which Predict Dietary Diversity and Meal Frequency Practice

Age of child in months was significantly associated (*P* ≤ 0.001) with dietary diversity. Children aged 12–17 and 18–23 months were 67% and 78% times less likely to practice adequate dietary diversity compared to children aged 6–11 months (OR = 0.322, 95% CI: 0.220, 0.472) and (OR = 0.215, 95% CI: 0.148, 0.313), respectively. Birth order is also found as important determinant for dietary diversity. Children who were born third had nearly two times more risk to be feed inappropriately compared to children born first (OR = 1.951, 95% CI: 1.152, 3.304).

Women with primary and secondary education were 67% and 70% less likely to practice inadequate dietary diversity ((OR = 0.314, 95% CI: 0.226, 0.438) and (OR = 0.296, 95% CI: 0.156, 0.562)) than those with no education. It was also learned from this study that having two children had 31% less chance of practicing adequate dietary diversity compared with having three children (OR = 0.690, 95% CI: 0.481, 0.992).

This study revealed that children born from the richest households had 74% less chance to have inadequate dietary diversity compared with children from the poorest household (OR = 0.256, 95% CI: 0.142, 0.459) (Table 2).

Age of child in month was found to be important predictor for meal frequency (*P* ≤ 0.001). Mothers with primary education and secondary and above education were 42% and 63% less likely to meet meal frequency inadequately compared with mothers with no education (OR = 0.579) and (OR = 0.364), respectively.

Exposure to media was significantly associated (*P* = 0.001) with meal frequency. Mothers with satisfactory exposure to media had 29% less risk to practice inadequate meal frequency compared to mothers with unsatisfactory exposure to media (OR = 0.707, 95% CI: 0.567, 0.882).

Numbers of antenatal care visits were significantly associated with minimum meal frequency (*P* = 0.004). Mothers with 4 and above ANC visits had 28% less risk to practice inadequate meal frequency compared with mothers with no ANC visit (OR = 0.720) (Table 3).

## 4. Discussion

Children with minimum dietary diversity score were found to be 10.8% which is very low when compared with results of eleven DHS reports of developing countries from Africa, Asia, and Latin America [15]. This may be due to poor food production and consumption as well as purchasing power of people in Ethiopia for variety of food items. Recent increment on price of consumable goods and poor knowledge preparation of complementary foods may contribute to inadequate dietary diversity in Ethiopian children.

The low dietary diversity coverage was almost similar to result obtained from revision of 2005 EDHS which was 7.1%. Prevalence of dietary diversity in our study was much lesser when compared with IYCF indicators in two African countries (37.4%) [16]. The dietary diversity in this study is similar to study done in India (15.2%) but lower than that Nepal (34%) and Bangladesh (41.9%) [17–19]. Poor economic status could be a reason for practicing inadequate dietary diversity in Ethiopian children [20].

Dietary diversity varies with age of infants and children. This finding is similar to a study in Bangladesh on revision of DHS 2007 [17]. Prevalence of low dietary diversity in age group 6–11 months in the present study is lower when compared with study conducted in Zambia and Ghana [16, 21]. It is also much lower when compared with south Asian countries like Bangladesh (81%), Nepal (82%), and Sri Lanka (88.3%) [17, 18, 22]. These might be due to poor feeding habit and limited household food availability in Ethiopian children.

The practice of meal frequency varies with age but there is improvement as the age increased. It was 37.4% and 66.9% in the first and third age group reported in Ethiopia, 72.7% and 99.7% in Ghana, and 66.7% and 90% in Bangladesh [17, 21], respectively.

According to this study, the most consistent predictors of dietary diversity were children's age in month, birth order, wealth index, number of children less than five year in the house, and mother education while predictors of meal frequency were maternal education, childs age in month, exposure to media, and ANC. A few previous studies also examined socioeconomic factors as predictors of dietary diversity and meal frequency practices in children [23]. The present study provides evidence for this association and reveals that poor maternal is education significantly associated with inadequate dietary diversity and meal frequency.

Age of child in months was important child characteristic which shows significant association both with dietary diversity and meal frequency. Infants aged 6–11 months were significantly less likely to meet those indicators. This result indicates the relationship between different food groups by age group which implies that food groups decrease as the child age decreases.

Low level of maternal education was associated with dietary diversity and meal frequency compared to those mothers who had secondary and higher levels of education which is in line with other study findings [17–19, 22], and it indicates that parental education plays a significant role in meeting the appropriate dietary diversity and meal frequency. In the long term, improvements in education leading to higher levels of parental education can result in better dietary diversity and meal frequency practices. In the short term, programs to improve those practices need to target families with low levels of parental education and design promotional materials that take account low parental levels of education. There is also evidence from the literature that the effect of maternal schooling on child nutritional status is conditioned by resource availability at the household level [6].

Media can be used as an effective means of promoting meal frequency practices. Women who had limited exposure to media showed an increased risk for suboptimal practices indicating the positive influence of media on feeding practice in Ethiopia as similar pattern has been observed in some countries [18, 19].

The number of antenatal care visits a mother had during her pregnancy was also related to meal frequency. A significantly higher risk of low meal frequency was observed among children whose mothers had no antenatal care visit. The fact that complementary feeding practice improved with exposure to health professionals could be due to the counseling they received from the health practitioner during the visit and they are likely to take appropriate actions to improve the dietary diversity of their children. Nutritional counseling for mothers about infant and young child feeding options during ANC is important continuum of care for promoting appropriate infant and young child feeding practice [18, 19, 24]. Birth order and number of children <5 year also remain important predictors of dietary diversity in this study. When the number of children <5 year is many, emphasis is given for increasing the variety of food given to children even if there is increased burden on mother to feed her children appropriately.

## 5. Conclusions

In conclusion, this study showed that a very low proportion of children aged 6–23 months in Ethiopia received adequate dietary diversity as measured by the WHO indicators. Inadequacy of dietary diversity is likely to negatively impact subsequent growth and development of Ethiopian children. Factors that consistently affect dietary diversity and meal frequency practice in common were age of the child especially lower age and low level of the mother's education. Birth order, wealth index, and number of children less than five years old were additional factors which determine minimum dietary diversity and exposure to media and ANC visit best predicts the minimum meal frequency.

## Figures and Tables

**Table 1:** Sociodemographic and other health related characteristics of children aged 6–23 months, Ethiopia, 2012.

Characteristics	Frequency	Percentage
Sociodemographic characteristics		
Husband education		
No education	1363	48.1
Primary education	1202	42.4
Secondary and above	237	8.4
Place of residence		
Urban	368	13
Rural	2468	87
Child characteristics		
Age in months		
6–11	1059	37.4
12–17	943	33.2
18–23	834	29.4
Birth order		
First	487	17.2
Second	500	17.6
Third	427	15.1
Fourth	371	13.1
Fifth	1051	37.1
Mothers characteristics		
Mothers work		
Yes	937	33.0
No	1894	66.8
Mothers education		
No education	1916	67.5
Primary	804	28.4
Secondary & above	116	4.1
Marital status		
Never in union	21	0.7
Married	2653	93.5
Widowed	162	5.7
Household characteristics		
Household wealth index		
Poorest	683	24.1
Poorer	630	22.2
Middle	571	20.1
Richer	507	17.9
Richest	445	15.7
Exposure to media		
Satisfactory	639	22.5
Unsatisfactory	2192	77.3
No. of children <5		
One	928	32.7
Two	1447	51.0
Three and above	461	16.3
Decision making		
Women involved	309	10.9
Women not involved	2341	88.3
Health service characteristics		
ANC visit		
No visit	1607	56.7
1–3	655	23.1
4+	494	17.4
Place of delivery		
Home	2503	88.3
Health institute	333	11.7

**Table 2:** Factors associated with minimum dietary diversity practice, Ethiopia, 2012.

Characteristics	Minimum dietary diversity		COR (95% CI)	AOR (95% CI)
	Adequate	Inadequate		
	*N* (%)	*N* (%)		
Child characteristics				
Age in month				
6–11 months	49 (4.6)	1010 (95.4)	1	
12–17 months	104 (11)	838 (89)	0.392 (0.276, 0.556)	0.322 (0.220, 0.472)
18–23 months	138 (16.5)	696 (83.5)	0.247 (0.176, 0.347)	0.215 (0.148, 0.313)
Birth order				
First	87 (17.9)	400 (82.1)	1	
Second	47 (9.4)	452 (90.6)	2.091 (1.432, 3.054)	2.134 (1.280, 3.557)
Third	41 (9.6)	387 (90.4)	2.084 (1.400, 3.102)	1.951 (1.152, 3.304)
Fourth	30 (8.1)	341 (91.9)	2.489 (1.603, 3.864)	2.110 (1.185, 3.757)
Fifth	87 (8.3)	87 (8.3)	2.433 (1.768, 3.348)	1.835 (1.127, 2.990)
Mothers characteristics				
Mother work status				
Yes	127 (13.6)	810 (86.4)	1.65 (1.292, 2.114)	1.271 (0.958, 1.688)
No	164 (8.7)	1730 (91.3)	1	
Mother education				
No education	105 (5.5)	1810 (94.5)	1	
Primary education	141 (17.5)	663 (82.5)	0.273 (0.209, 0.357)	0.314 (0.226, 0.438)
Secondary and above	45 (38.8)	71 (61.2)	0.090 (0.059, 0.138)	0.296 (0.156, 0.562)
Marital status				
Never in union	3 (14.3)	18 (85.7)	1	
Married	283 (10.7)	2369 (89.3)	1.212 (0.332, 4.419)	∗∗
Widowed	5 (3.1)	157 (96.9)	4.191 (0.893, 19.680)	∗∗
Household characteristics				
Household wealth index				
Poorest	34 (5)	649 (95)	1	
Poorer	60 (9.5)	570 (90.5)	0.492 (0.318, 0.761)	0.635 (0.402, 1.004)
Middle	39 (6.8)	533 (93.2)	0.717 (0.446, 1.152)	1.006 (0.610, 1.658)
Richer	44 (8.7)	463 (91.3)	0.552 (0.347, 0.877)	0.923 (0.560, 1.521)
richest	115 (25.8)	330 (74.2)	0.150 (0.100, 0.226)	0.256 (0.142, 0.459)
Exposure to media				
Satisfactory	172 (7.8)	2020 (92.2)	0.372 (0.289, 0.478)	0.734 (0.531, 1.014)
Unsatisfactory	119 (18.6)	520 (81.4)	1	1
Number of <5 children				
one	124 (13.4)	804 (86.6)	1.155 (0.806, 1.656)	1.880 (1.251, 2.826)
Two	123 (8.5)	1324 (91.5)	0.690 (0.481, 0.992)	2.476 (1.510, 4.060)
Three and above	44 (9.6)	416 (90.4)	1	0.455 (0.284, 0.729)
Decision making				
Women not involved	26 (8.4)	282 (91.6)	1	
Women involved	257 (11.0)	2084 (89)	1.323 (0.869, 2.012)	∗∗
Sociodemographic characteristics				
Husband education				
No education	84 (6.2)	1279 (93.8)	1	
Primary education	137 (11.4)	1065 (88.6)	0.510 (0.384, 0.678)	0.944 (0.681, 1.308)
Secondary and above	65 (27.4)	172 (72.6)	0.174 (0.121, 0.249)	0.804 (0.474, 1.365)
Type of place of residence				
Urban	83 (22.6)	285 (77.4)	3.162 (2.384, 4.195)	0.687 (0.410, 1.152)
Rural	208 (8.4)	2260 (91.6)	1	
Health care characteristics				
Number of ANC visits				
No visit	116 (7.2)	1491 (92.8)	1	
1–3 visits	75 (11.5)	580 (88.5)	0.600 (0.442, 0.815)	0.903 (0.640, 1.275)
4+ visits	100 (20.2)	394 (79.8)	0.307 (0.229, 0.410)	0.749 (0.511, 1.098)
Place of delivery				
Home	216 (8.6)	2287 (91.4)	1	
Health institute	75 (22.6)	257 (77.4)	3.093 (2.309, 4.143)	1.229 (0.753, 2.006)

OR: odds ratio; CI: confidence interval.

**Those not showing association in the Bivariate analysis at *P* value ≤0.2 and not fitted to multivariate model.

**Table 3:** Factors associated with minimum meal frequency practice, 2012, Ethiopia.

Characteristics	Minimum meal frequency		COR (95% CI)	AOR (95% CI )
	Adequate	Inadequate		
	*N* (%)	*N* (%)		
Child characteristics				
Age in month				
6–11 months	384 (37.4)	643 (62.6)	1	
12–17 months	454 (51.8)	454 (51.8)	1.67 (0.591, 1.720)	1.422 (1.233, 1.870)
18–23 months	220 (33.1)	220 (33.1)	1.580 (0.580, 1.701)	1.211 (1.010, 1.502)
Birth order				
First	228 (52.9)	203 (47.1)	1	
Second	225 (49.5)	230 (50.5)	1.148 (0.882, 1.494)	1.019 (0.766, 1.356)
Third	172 (44.4)	215 (55.6)	1.401 (1.063, 1.845)	1.304 (0.966, 1.759)
Fourth	150 (42.6)	202 (57.4)	1.513 (1.139, 2.009)	1.198 (0.878, 1.634)
Fifth	475 (50.4)	468 (49.6)	1.109 (0.883, 1.394)	0.863 (0.667, 1.116)
Mothers characteristics				
Mother work status				
Yes	457 (53.8)	393 (46.2)	1.36 (0.921, 1.411)	1.202 (0.997, 1.448)
No	790 (46.1)	924 (53.9)	1	
Mother educational status				
No education	785 (44.5)	981 (55.5)		
Primary education	398 (56.6)	305 (43.4)	0.632 (0.526, 0.758)	0.579 (0.465, 0.720)
Secondary and above	68 (68)	32 (32)	0.401 (0.275, 0.585)	0.364 (0.202, 0.654)
Marital status				
Never in union	10 (52.6)	9 (47.4)	1	
Married	1162 (48.5)	1236 (51.5)	1.160 (0.478, 2.818)	∗∗
Widowed	79 (52.3)	72 (47.7)	0.986 (0.385, 2.524)	∗∗
Household characteristics				
Wealth index				
Poorest	268 (42.4)	364 (57.6)	1	
Poorer	275 (48.0)	298 (52.0)	0.799 (0.637, 1.004)	0.873 (0.687, 1.110)
Middle	262 (51.4)	248 (48.6)	0.695 (0.550, 0.879)	0.860 (0.669, 1.104)
Richer	240 (52.2)	220 (47.8)	0.674 (0.530, 0.864)	0.902 (0.692, 1.176)
Richest	206 (52.3)	188 (47.7)	0.671 (0.521, 0.864)	1.083 (0.770, 1.524)
Exposure to media				
Satisfactory	916 (46.1)	1071 (53.9)	0.888 (0.804, 0.971)	0.707 (0.567, 0.882)
Unsatisfactory	332 (57.5)	345 (42.5)	1	
Number of <5 children				
One	444 (51.4)	420 (48.6)	1.036 (0.822, 1.306)	∗∗
Two	631 (47.2)	707 (52.8)	0.876 (0.686, 1.118)	∗∗
Three and above	176 (48.1)	190 (51.9)	1	
Decision making				
Women not involved	121 (43.4)	158 (56.6)	1	
Women involved	1039 (49.1)	1078 (50.9)	0.793 (0.616, 1.020)	1.144 (0.861, 1.519)
Sociodemographic characteristics				
Husband education				
No education	570 (45.7)	676 (54.3)	1	
Primary education	548 (50.6)	534 (49.4)	0.821 (0.698, 0.967)	1.049 (0.871, 1.263)
Secondary and above	116 (55.2)	94 (44.8)	0.685 (0.510, 0.919)	1.359 (0.917, 2.013)
Type of place of residence				
Urban	166 (50.6)	162 (49.4)	1.087 (0.862, 1.371)	∗∗
Rural	1085 (48.4)	1155 (51.6)	1	
Health care characteristics				
Number of antenatal visits				
No visit	673 (44.7)	834 (55.3)	1	
1–3 visits	308 (50.7)	299 (49.3)	0.783 (0.648, 0.945)	0.923 (0.745, 1.143)
4+ visits	236 (61)	151 (39)	0.544 (0.440, 0.674)	0.720 (0.549, 0.946)
Place of delivery				
Home	1084 (47.8)	1184 (52.2)	1	
Health institution	167 (55.7)	133 (44.3)	1.364 (1.071, 1.738)	0.989 (0.697, 1.402)

OR: odds ratio; CI: confidence interval. **Those not showing association in the crude odds ratio at *P* value ≤0.2.

## Abbreviations

BMI: Body mass index
CSA: Central Statistical Agency
DHS: Demographic and Health Survey
EDHS: Ethiopian Demographic and Health Survey
IYCF: Infant and young child feeding
MDD: Minimum dietary diversity
MMF: Minimum meal frequency
MDG: Millennium Development Goal
SPSS: Statistical package for social science
WHO: World Health Organization.
